# Cervical Spondylotic Myelopathy Caused by Single-Level Vertebral Spontaneous Fusion

**DOI:** 10.1371/journal.pone.0112423

**Published:** 2014-11-07

**Authors:** Ning Yan, Shunzhi Yu, Tiesheng Hou, Guangfei Gu, Hailong Zhang, Shan Zhao, Shisheng He

**Affiliations:** Department of Orthopedic Surgery, Shanghai 10th People's Hospital, Tongji University School of Medicine, Shanghai, China; University of Michigan, United States of America

## Abstract

**Purpose:**

To evaluate the clinical features, imaging characteristics, surgical options, and clinical outcomes of patients with Cervical spondylotic myelopathy (CSM) caused by single-level vertebral spontaneous fusion (SLVSF).

**Methods:**

Sixteen consecutive patients with SLVSF who underwent anterior surgery were included in this study and 38 patients with CSM caused by spinal degeneration were enrolled as a control group. Demographic features, clinical presentations, imaging characteristics, surgery strategy, Nurick grade, Japanese Orthopedic Association (JOA) score, neck disability index (NDI), and complications were evaluated.

**Results:**

There were significant differences between the two groups in the mean age and the average duration of neck pain. There was no significant difference between the two groups in length of cervical spine. In the SLVSF group, 13 patients had upper segment translational instability and none had rotational instability. Pre- and postoperative Nurick grades were 2.94±0.77 and 2.19±0.54 in the SLVSF group, and 2.97±0.72 and 2.16±0.64 in the control group. Pre- and postoperative JOA scores were 9.25±2.02 and 11.69±1.62 in the SLVSF group, and 9.87±2.58 and 12.53±2.69 in the control group. Pre- and postoperative NDI values were 28.5±7.75 and 15.56±5.51 in the SLVSF group, and 16±6.13 and 11.29±4.58 in the control group.

**Conclusions:**

Patients with SLVSF have necks of normal lengths, which can be used to distinguish this disorder from Klippel-Feil syndrome. There are three main features of SLVSF: (1) hypoplasia at both of the spontaneously fused vertebral bodies; (2) a major pathological feature of translational instability of the upper vertebra to the fused level; and (3) severe neck pain. Anterior surgery has a good therapeutic effect for patients with cervical SLVSF.

## Introduction

Cervical spondylotic myelopathy (CSM) is the most common cause of spinal cord dysfunction in the elderly [1]. Typically caused by spinal degeneration, cervical congenital malformations such as developmental stenosis may predispose to CSM. In addition to congenital cervical spinal stenosis [2], spontaneous fusion of the cervical vertebrae is a type of congenital malformation that is also a known cause of CSM. However, to the best of our knowledge, this type of CSM has not been described in detail. From 2002 to 2012, we treated 16 patients with CSM because of single-level vertebral spontaneous fusion (SLVSF) of the cervical vertebrae in our hospital. Here, we summarized the diagnosis and treatment of patients with SLVSF compared to those of patients with CSM caused by spinal degeneration.

## Materials and Methods

This retrospective study was approved by the Ethical Committee of Shanghai 10th People's Hospital. All participants gave their informed written consent for publication of their medical documents and images to the Ethical Committee of Shanghai 10th People's Hospital.

### Patients

The survey pertained to all CSM patients who had undergone anterior surgery from July 2002 to June 2012 in our institution. According to the lateral X-ray, we collected 23 patients with cervical vertebra body fusion. Patients with cervical spinal trauma, tumors, tuberculosis, infection, surgery, ossification of the posterior longitudinal ligament (OPLL), upper cervical deformity, developmental cervical spinal stenosis, or incomplete records were excluded. Finally, a total of 16 patients (11 women and 5 men; median age, 48.5±4.9 years; age range, 41–63 years) were included in this study as a SLVSF group. Radiological diagnoses were established in each patient via routine preoperative cervical anteroposterior, lateral, and flexion-extension radiographs and cervical magnetic resonance imaging (MRI) or computed tomography (CT) scans. The level of spontaneously fused segments, spinal cord segments with lesions, and surgical segment were recorded.

Patients diagnosed with CSM caused by spinal degeneration and underwent anterior cervical surgery between 2010 and 2012 were included as a control group. The extent of compression was no more than three segments in all patients. Besides the exclusion criteria of the SLVSF group, those who underwent cervical artificial disc implantation were also excluded. Of the 134 patients with CSM caused by spinal degeneration and underwent anterior cervical surgery, 96 patients were excluded and 38 patients were included in this study.

All surgeries were performed by a single surgeon (H.T.) with more than 30 years of clinical experience in cervical spine surgery. And all the clinical decisions were made according to standard care for the given medical conditions. The following data were recorded for each patient: history; symptoms on admission; duration of symptoms; physical and neurological findings at presentation; intraoperative spinal observations; preoperative and 3-, 6-, and 12-month postoperative neurological function; preoperative and 3-, 6-, and 12-month postoperative radiological findings, intra- and postoperative complications; and follow-up duration.

### Surgical Indications

Compression of the cervical spinal cord was confirmed on MRI or CT. Segmental instability was evaluated on dynamic lateral radiographs. All patients received conservative treatment preoperatively, which consisted of analgesics and muscle relaxants, cervical collar protection, bed rest, and appropriate physical therapy. The indication for surgery was cervical myelopathy followed by long-term incapacitating pain, radiculopathy, segmental instability, and radiculomyelopathy.

### Outcome Measures

**Figure 1:**
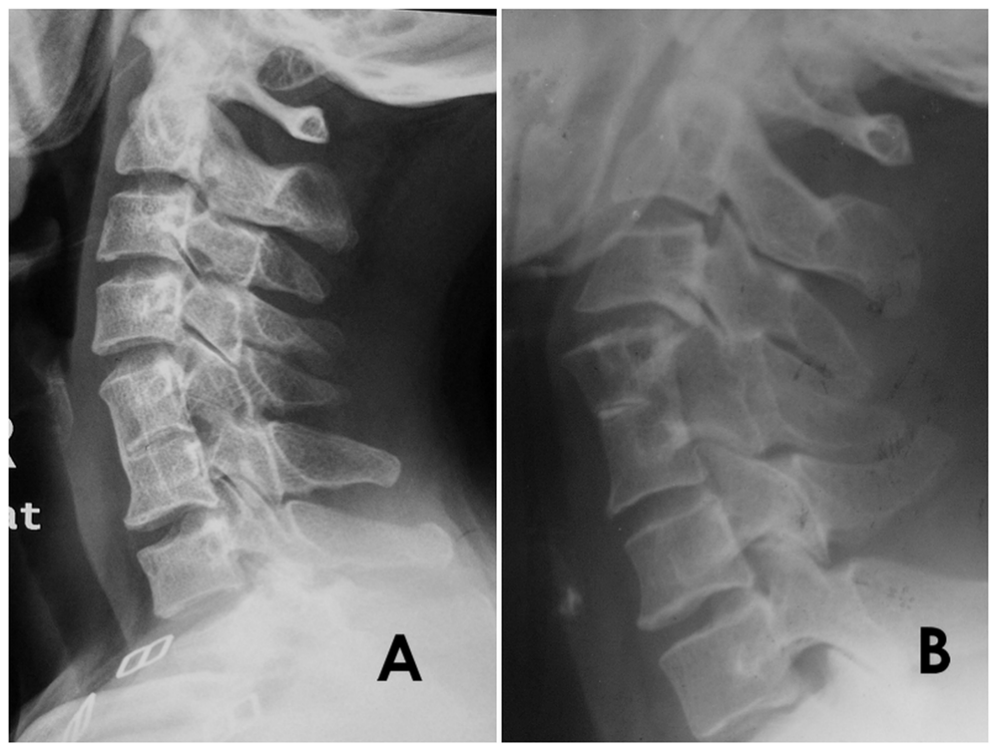
Lateral view of cervical single-level vertebral spontaneous fusion. A, Fusion of the vertebral bodies and the zygapophyseal joints, where the spinous processes remained independent; B, Fusion of the vertebral bodies, the zygapophyseal joints, and the spinous processes.

All patients underwent radiography, MRI, and CT preoperatively. We judged the level and range of spontaneous fusion by lateral X-rays. Patients were divided into two groups according to the range of spontaneous fusion: type I, indicated by fusion of the vertebral bodies and the zygapophyseal joints, where the spinous processes remained independent; and type II, indicated by fusion of the vertebral bodies, the zygapophyseal joints, and the spinous processes (Fig.1A, B).

**Figure 2:**
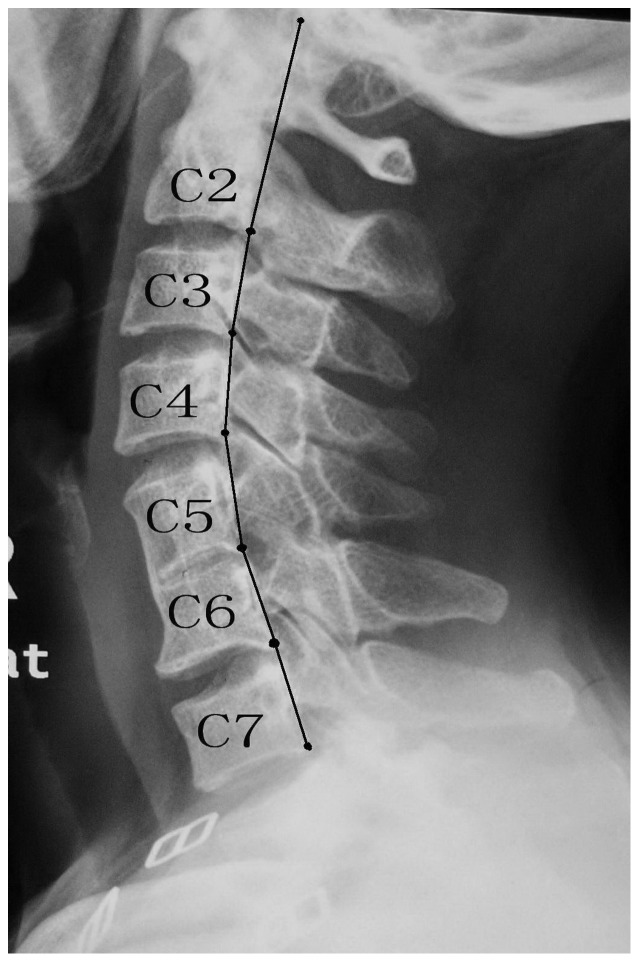
Measurements of cervical spine lengths were made by locating seven points and connecting each point in-sequence.

We compared the mean cervical spine lengths between the two groups. All measurements were made by locating seven points and connecting each point in-sequence. The seven points included the point of intersection of an extended line of the C2 vertebral posterior marginal and occipital and the lower points of the posterior margin of the C2, C3, C4, C5, C6, and C7 vertebral bodies. From these points, we derived six line segments and defined the cervical length as the total length of all six line segments (Fig.2).

**Figure 3:**
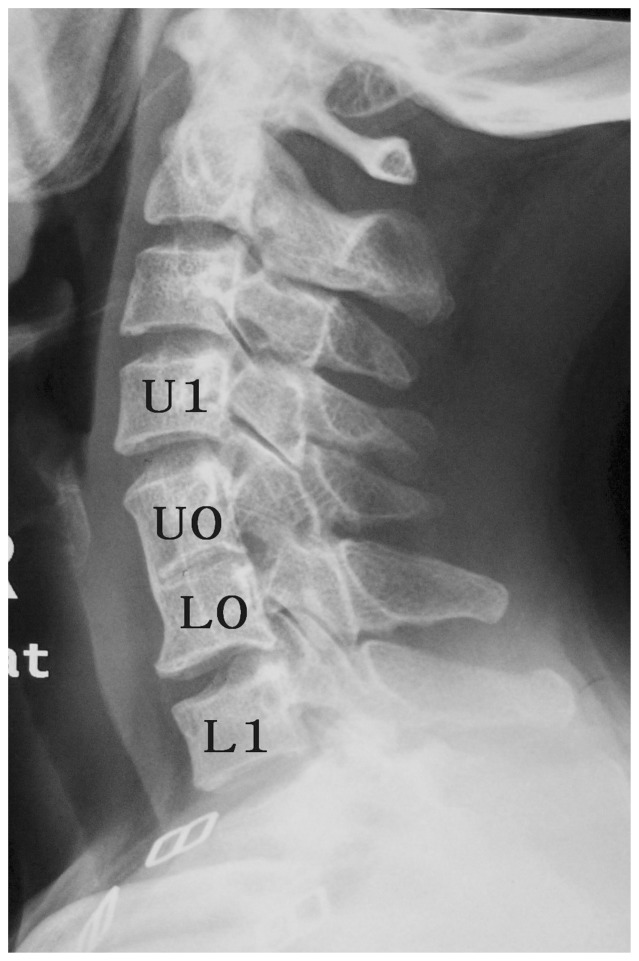
Definitions of the fused vertebrae and adjacent vertebrae.

**Figure 4:**
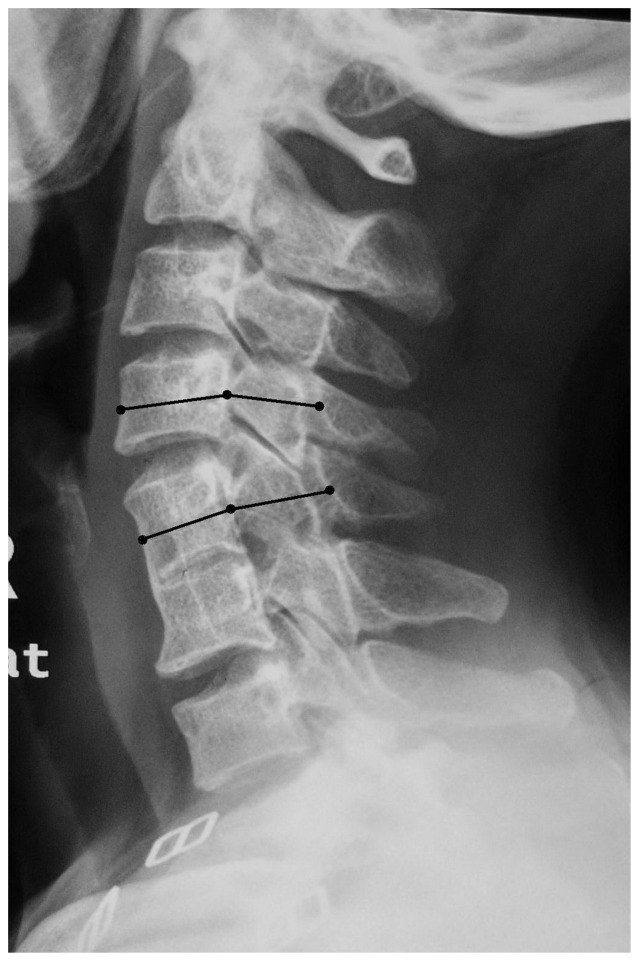
Spinal canal index is equal to the sagital diameter of the spine canal divided by the anterior-posterior length of the body, at its mean point.

We measured the spinal canal index from C3 to C7 of all patients. In the SLVSF group, two adjacent vertebral were fused. We defined the upper vertebral body as U0 and the lower vertebral body as L0. Also, we defined adjacent cephalic vertebrae of U0 as U1 and the adjacent caudal vertebra of L0 as L1(Fig.3). The measurement method of spinal canal index is shown in Fig.4 [3], [4].

**Figure 5:**
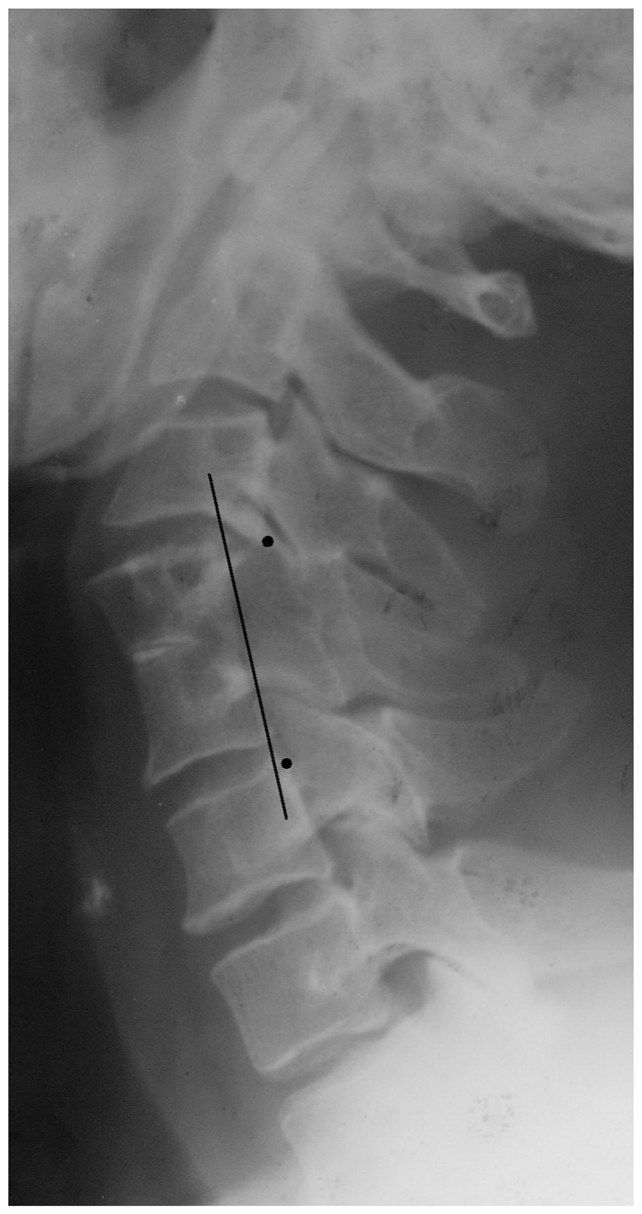
The translational instability of the adjacent vertebra was measured as the distance between the superior/inferior point of the posterior L1/U1 vertebral body from the extended line of the posterior U0-L0 vertebral margin.

Segmental instability was determined by flexion-extension lateral radiographs according to the White–Panjabi standard: (1) translational instability, characterized by horizontal displacement of one vertebra of more than 3.5 mm in relation to an adjacent vertebra, and (2) rotational instability, characterized by rotational difference between adjacent vertebrae of more than 11 degrees [5]. In the SLVSF group, because the extended lines from the posterior vertebral marginal of U1 and U0 were crossed, we were unable to measure the distance of the two cross-straight lines, thus we made a slight modification. The translational instability of the adjacent cephalic vertebra was defined as TIU, which was measured as the distance between the inferior point of the U1 posterior vertebral body from the extended line of the U0-L0 posterior vertebral margin. The translational instability of the adjacent caudal vertebra was defined as TIL, which was measured as the distance between the superior point of the posterior L1 vertebral body from the extended line of the posterior U0-L0 vertebral margin (Fig.5). The rotational instability of U1 was defined as RIU and that of L1 was defined as RIL. The measurement methods refer to White A.A. [5].

Cervical anteroposterior, lateral, and flexion-extension radiographs were obtained during follow-up examinations performed at 3, 6, and 12 months postoperatively. Neurological function was assessed using the Nurick classification system for myelopathy (Nurick grade) and the Japanese Orthopedic Association scoring system for the evaluation of cervical myelopathy (JOA score). The rate of improvement was calculated using the following formula: [(postoperative JOA score-preoperative JOA score)/(17-preoperative JOA score)]×100%. Surgical outcome was defined according to the improvement rate as follows: excellent (improvement rate > 75%), good (75% >improvement rate >50%), fair (50% >improvement rate >25%), and poor (improvement rate <25%). The neck disability index (NDI) was also recorded for the evaluation of neck-shoulder pain levels. Since driving is not essential in China, we deleted section 8: Driving.

### Statistical Analysis

Data were analyzed using Microsoft Excel 2003 (Microsoft, Redmond, WA, USA) and SPSS version 19.0 software (SPSS, Inc., Chicago, IL, USA). Paired data were compared using a paired t-test, where unpaired data were compared using an independent t-test. The chi-squared test was used for categorical data. A probability (p) value <0.05 was considered statistically significant.

## Results

Patients were followed for 12 to 40 months postoperatively. Of the 16 patients in the SLVSF group, 68.8% (11) were female and of the 38 patients in the control group, 44.7% (17) were female. There was a significant difference in mean age between the SLVSF and control groups (48.5±4.9 vs. 57.2±6.4 years, respectively). The demographic information and follow-up of two groups are shown in Table 1. The distribution of spontaneously infused segments was as follows: C3–4, n = 2 patients; C4–5, n = 5; C5–6, n = 7; and C6–7, n = 2. In the SLVSF group, five patients had type I spontaneous fusion and eleven had type II spontaneous fusion. The average duration of neck pain was 15.8±3.2 and 3.4±0.6 years in the SLVSF and control groups, respectively. The average duration of cervical myelopathy was 14±4.4 years and 22.8±8.8 months in the SLVSF and control groups, respectively. There was no significant difference in the length of the cervical spine between the SLVSF and control groups (female/male, 13.88±0.45/14.46±0.69 cm vs. 13.87±0.36/14.61±0.48 cm, respectively).

**Table 1:** Demographic information and follow-up of two groups.

	SLVSF(n = 16)	Control(n = 38)	p value
Age(mean±SD)	48.5±4.9	57.2±6.4	<0.001
Gender n(%)			
Male	5(31.2%)	21(55.3%)	
Female	11(68.8%)	17(44.7%)	
Type			
I	5		
II	11		
Follow-up(months)	17.4±3.9	20.3±4.5	0.80

SLVSF: single-level vertebral spontaneous fusion.

### Spinal canal Index

The mean spinal canal indices were as follows: (SLVSF group) U1,0.96±0.08; U0, 1.06±0.07; L0,1.07±0.08; L1, 0.93±0.07 and (control group) C3, 0.91±0.09; C4, 0.92±0.07; C5, 0.92±0.08; C6, 0.96±0.07; C7, 0.97±0.06?There was no significant difference in spinal canal index between U0 and L0. The spinal canal index of U0 and L0 was significantly higher than that of U1, L1, and any segment in the control group.

### Vertebral Instability

In the SLVSF group, the average vertebral instability of TIU, TIL, RIU, and RIL was 3.64±0.58 mm, 0.44±0.63 mm, 3.56°±2.48, and 2.31°±1.45, respectively. According to the White–Panjabi standard, 13 patients had U1 translational instability, but none had L1 translational instability, and no patient exhibited rotational instability in the SLVSF group. In the control group, three patients with three segments exhibited translational instability, but none exhibited rotational instability.

### Surgical Strategy

**Figure 6:**
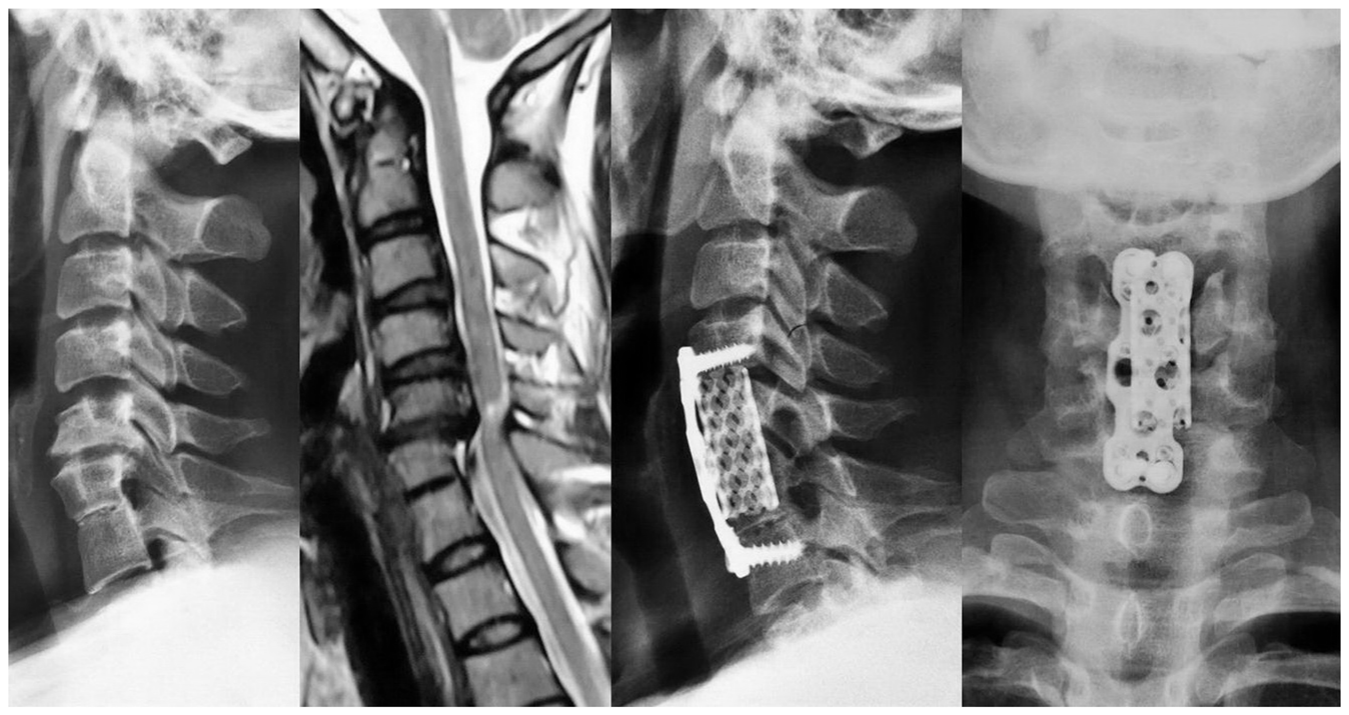
Anterior cervical corpectomy and fusion, the corpectomy encompassed the fused upper vertebral body and the superior part of the fused lower vertebral body.

All patients in the SLVSF group underwent anterior cervical vertebral (U0) corpectomy and received titanium mesh implants using internal fixation procedures. The corpectomy encompassed the fused upper vertebral body and the superior part of the fused lower vertebral body and was performed using the Slimlock Anterior Cervical Plate System (DePuy Synthes Spine, Inc., Raynham, MA, USA) (Fig.6). In the control group, four patients underwent anterior cervical discectomy and fusion, 24 underwent cervical corpectomy with titanium mesh implants and internal fixation, and 10 underwent anterior cervical corpectomy and discectomy with fusion and fixation (Slimlock Anterior Cervical Plate System).

The functional results are shown in Table 2. No significant intergroup differences in Nurick grades or JOA scores were observed preoperatively. The NDI of the SLVSF group was higher than that of the control group both pre- and postoperatively. The Nurick grade, JOA score, and NDI significantly improved in both groups postoperatively. The improvement in JOA score of the SLVSF group was excellent in 8 patients, good in 5, fair in 3, and poor in 0, and excellent in 14 patients, good in 17, fair in 5 and poor in 2 in the control group.

**Table 2:** Function Scores between Groups.

Clinical Outcomes between Groups			
	Mean±SD		
Variable	SLVSF(n = 16)	Control(n = 38)	p value
Nurick grade			
Preoperative	2.94±0.77	2.97±0.72	0.89
Postoperative	2.19±0.54	2.16±0.64	0.87
JOA score			
Preoperative	9.25±2.02	9.87±2.58	0.40
Postoperative	11.69±1.62	12.53±2.69	0.25
NDI			
Preoperative	28.5±7.75	16±6.13	<0.001
Postoperative	15.56±5.51	11.29±4.58	0.005

SLVSF: single-level vertebral spontaneous fusion. JOA score: Japanese Orthopedic Association score. NDI: Neck Disability Index.

A summary of complications is shown in Table 3. No cases of infection, chronic inflammation, iatrogenic neurological deterioration, or hematoma occurred during the follow-up period.

**Table 3:** Complications.

	SLVSF Group	Control Group
Axial neck pain	11	5
Temporary odynophagia	2	5
C5 root palsy	0	2

SLVSF: single-level vertebral spontaneous fusion.

## Discussion

Cervical vertebral spontaneous fusion is a rare cervical congenital malformation [6]–[8]. Most cases of cervical congenital malformations are reported as Klippel–Feil syndrome. In 1912, Maurice Klippel and Andre Feil independently provided the first descriptions of Klippel–Feil syndrome, in which patients are characterized by a short, webbed neck, decreased range of motion (ROM) in the cervical spine, and a low hairline. Feil subsequently classified the syndrome into three categories: type I (fusion of C2 and C3 with occipitalization of the atlas), type II (long fusion below C2 with an abnormal occipital-cervical junction), and type III (a single open interspace between two fused segments, cervical spine motion is concentrated at single open articulation). Cervical SLVSF below C2 is not included in this definition.

In 2006, Samartzis et al. [9] proposed three classification types that specifically addressed cervical spine anomalies and their associated cervical spine-related symptoms, with additional elaboration on various time-dependent factors regarding this syndrome. Radiographically, type I is defined by the presence of a single congenitally fused cervical segment, where type II is characterized by multiple noncontiguous, congenitally fused segments, and type III by multiple contiguous, congenitally fused cervical segments. According to this definition, the cases of SLVSF in our study were of type I. However, a primary characteristic of Klippel–Feil syndrome is a short neck. In our study, there was no difference in the cervical spine length between the two groups. The patients in our study did not display this characteristic feature of Klippel–Feil syndrome; therefore, we were able to distinguish this congenital malformation from Klippel–Feil syndrome.

In regard to pathogenesis of spontaneous fusion, Gunderson et al. [10] considered this abnormality to be a defect resulting from non-segmentation of the sclerotomes. Gray et al. [11] were of the opinion that chondrification of the disc tissue led to fusion of the vertebrae, whereas Chandraraj [8] considered that non-development of the joint between articular facets results in fusion of the vertebral arches, which, in turn, leads to secondary fusion of the bodies and hypoplasia of the intervertebral discs. We are of the opinion that there were two kinds of SLVSF exhibited by the participants in this study according to the lateral X-rays. The first type involved fusion of the vertebral bodies, the zygapophyseal joints, and the spinous processes. We contend that fusion of these three positions occurred simultaneously because of non-segmentation of the sclerotomes. In the second type, the vertebral bodies and zygapophyseal joints were fused, but the spinous processes remained independent. The most likely reason for this fusion is disc hypoplasia in advance of vertebral fusion. Because of the loss of motor function of the fused segments, the zygapophyseal joints fused secondarily. A radiographic image of one case (Fig.1A) showed that vertebral bodies were completely fused and there was a faint remnant space in the zygapophyseal joints. Thus, it could be assumed that the zygapophyseal joints fused secondarily to the vertebral bodies. However, these points are just conjecture. To make a precise conclusion, it would be necessary to discern the entire natural history of SLVSF. Unfortunately, there is currently no correlative report.

Regardless of the reason of fusion, there was no significant difference in age of onset, course of disease, degree of cervical instability, or pre- and postoperative neurological function among patients in the two groups. Both types of fusion led to the loss of motion of the fused segments and excessive activity of adjacent segments, which could ultimately lead to degeneration of the adjacent segments. However, this pathological process is largely determined by vertebral fusion and whether the zygapophyseal joints and spinous processes were fused made no difference. Therefore, this classification scheme only has significance in regard to imaging and no significance in clinical guidance.

In our study, there were significantly more females than males in the SLVSF group, as with the study by Samartzis et al. [9]. However, the reason for the larger number of females remains unclear.

The results of this study indicated that the major cause of cervical instability was the translational instability of the upper vertebrae to the fused level (U1 to U0). The U1 vertebra had a tendency to shift more posteriorly at the position of cervical hyperextension. However, the rotational stability remained normal and there was no instability between L1 and L0. We consider that the translational instability of U1 was the key factor leading to spinal cord compression. The main cause of cervical instability was spontaneous fusion, which induced stress on the adjacent segments. This could also explain the younger age of patients in the SLVSF group. Most patients reported neck pain for 5–20 years.

The reason for the lack of instability at the lower segment can be explained by the increase in size of the inferior vertebral bodies compared to superior vertebrae; therefore, the ability to withstand stress is greater in the lower segment than the upper segment. When stress exceeds the capacity of the intervertebral discs, instability occurs first in the upper segment. A vicious circle of stress concentration-instability-degeneration forms and reinforces itself, in which the lower segments bear less stress during movement. Bydon [12] reported that patients were more likely to develop adjacent segment disease (ASD) above the index level of fusion in a study of ASD after anterior cervical discectomy and fusion. The results of previous biomechanical experiments are also consistent with these findings.

Congenital cervical spinal stenosis is among the pathogenic factors of CSM. However, our results showed that the values of spinal canal indices were greater at the spontaneous fused segments than those at the other segments. The vertebral bodies of spontaneously fused segments at the sagittal plane are relatively small. Meanwhile, the vertebral bodies take on the shape of an hourglass, marked by noticeable thinning at the midline. Although the sagittal length of the spinal canal remained unchanged, the spinal canal index became larger because of the smaller numerator. Our findings demonstrated that in patients with SLVSF, hypoplasia exists at both of the involved vertebral bodies and intervertebral discs. These characteristics may have a significant effect on the choice of surgery.

Several factors should be considered when deciding on a surgical method for patients with SLVSF. First, spinal cord compression is usually located at the U1/U0 level because there is obvious instability at this level, so it is inappropriate to adopt anterior cervical discectomy with arthroplasty. Second, the vertebral body of U0 has a slim mid portion and the sagittal length is relatively short. If we were to perform anterior discectomy and fusion, two screws are placed in the middle of the vertebral body. However, only shorter screws can be used at this location, resulting in limited anchoring force. We adapted anterior corpectomy and fusion, in which the vertebral bodies of U0 and superior part of L0 were resected. With appropriate distraction, the translational displacement was redressed. After inserting titanium mesh, fixation was performed with a plate and screws. Two screws were placed in the upper portion of the L1 vertebral body. Screws with a length of 12–14 mm can provide sufficient anchoring force.

The follow-up results showed that most patients in the SLVSF group had improved neurological function of good to excellent. However, there was no significant difference in the Nurick grade or JOA score between the SLVSF and control groups. The NDI was designed to assess the effect of neck pain on the ability to perform everyday tasks. NDI values were higher in the SLVSF group than the control group both pre- and postoperatively, indicating that neck pain is the prominent symptom in patients with SLVSF, and it was not totally alleviated after surgery.

Three types of complications occurred in all patients: axial neck pain, temporary odynophagia, and C5 root palsy. Axial pain refers to pain in the shoulders, back, and neck caused by cervical spondylosis [13]. The incidence of axial neck pain was higher is the SLVSF group than the control group, which was consistent with the higher NDI values and longer duration in the SLVSF group. Yoshida [14] noted that postoperative axial neck pain was likely to occur if patients had preoperative neck pain. Early and sharp axial neck pain is a characteristic symptom of SLVSF.

There was no difference in the incidence of temporary odynophagia between the two groups. There were two cases of C5 root palsy in the control group and none in the SLVSF group. Two hypotheses were formulated for the cause of C5 root palsy. One hypothesis is that C5 palsy is caused by nerve root damage. Others have presumed that tethering of the nerve roots might cause C5 palsy as the result of a shift of the spinal cord in association with anchoring of the nerve root [15]–[17]. Each patient with SLVSF had a voluminous spinal canal, thus direct damage to the nerve root or a shift of the spinal cord seldom occurred. There was no C5 root palsy observed among patients in the SLVSF group.

## Conclusions

SLVSF is a congenital malformation which can predispose to CSM. Patients with SLVSF have cervical spines of a normal length, which can be used to distinguish this disorder from Klippel-Feil syndrome. There are three distinct features of SLVSF: (1)hypoplasia of both spontaneously fused vertebral bodies and intervertebral discs; (2) a major pathological feature of translational instability of the upper vertebral to the fused level, whereas the upper vertebrae have a tendency to shift more posteriorly at the position of cervical hyperextension; and (3) severe neck pain, which is rarely totally alleviated. As an appropriate surgery, we recommend anterior corpectomy and fusion, where the corpectomy extends from the fused upper vertebral body to the superior part of the fused lower vertebral body. Anterior surgery has a good therapeutic effect for patients with cervical SLVSF. There was no difference in the Nurick grade or JOA score between the SLVSF and control groups. The NDI was higher in the SLVSF group than the control group both pre- and postoperatively.
